# Dose-specific effects of caffeinated chewing gum on reactive blocking timing, jump mechanics, and neuromuscular responses after volleyball-specific fatigue in elite female volleyball players

**DOI:** 10.1080/15502783.2026.2749890

**Published:** 2026-11-09

**Authors:** Jinghui Zhong, Jiyan Xu, Chang Lu, Hao Wu

**Affiliations:** a Capital University of Physical Education and Sports, Beijing, China; b Beijing University of Chinese Medicine, Beijing, China

**Keywords:** Caffeine, volleyball, fatigue, block jump, female athletes

## Abstract

**Purpose:**

To investigate the effects of caffeinated chewing gum at nominal target doses of 3 and 6 mg/kg on reactive blocking timing, block-jump mechanics, and neuromuscular responses in elite female volleyball players under post-fatigue conditions.

**Methods:**

Fifteen elite female volleyball players completed a randomized, placebo-controlled crossover study implemented with double-blinding procedures. Participants consumed placebo (PLA), caffeinated chewing gum at nominal target doses of 3 mg/kg (CAF3) or 6 mg/kg (CAF6), and were tested after supplementation and warm-up at PRE and immediately after a volleyball-specific repeated blocking fatigue protocol (POST). Primary outcomes were reactive blocking timing-chain indices and block-jump (BJ) peak power. Secondary outcomes included additional BJ mechanical variables and general jump measures from countermovement jump (CMJ) and drop jump (DJ) tests. Exploratory outcomes included synchronized surface electromyography (sEMG) variables of lower-limb muscles. Participant blinding was assessed after each visit.

**Results:**

Fatigue impaired jump-related performance across conditions (*p* < 0.05). For the primary outcomes, the descriptive decline in BJ peak power was 6.9% in CAF6 versus 13.9% in PLA; the adjusted POST comparison did not establish superiority of CAF6 over CAF3. For timing outcomes, overall sequence time increased by 18.4% in PLA, 6.4% in CAF3, and 3.0% in CAF6; CAF6 therefore showed the smallest descriptive deterioration in the integrated sequence measure, whereas CAF3 showed little change in the mean stationary and movement component times. CAF6 showed a reduction in mean stationary response time and the smallest increase in overall sequence time. For secondary outcomes, caffeine showed more favorable post-fatigue responses in selected CMJ/DJ measures, including CMJ mean propulsive power and DJ contact time (*p* < 0.05), and CAF6 showed greater preservation of selected stretch-shortening cycle-related mechanical measures, including DJ jump height and DJ vertical stiffness, than PLA (*p* < 0.05). In contrast, CMJ mRSI showed a divergent pattern and was not interpreted as evidence of caffeine-related attenuation of fatigue-induced decrements. Exploratory sEMG results showed smaller post-fatigue changes in selected frequency-domain and activation-timing variables under CAF6, particularly in measures related to extensor-muscle function. Participant masking was incomplete: perceived caffeine ingestion was reported in 0.0%, 53.3%, and 80.0% of PLA, CAF3, and CAF6 visits, respectively, and adverse effects were more frequent in CAF6.

**Conclusions:**

The observed responses were outcome-specific: CAF6 showed a smaller decline in BJ peak power and the smallest increase in overall sequence time, whereas CAF3 showed little change in mean stationary and movement response times. These findings do not establish a generally preferable dose or a timing-stability advantage for CAF3. Incomplete participant masking limits attribution of the differences exclusively to caffeine dose.

## 1. Background

Volleyball features repeated offensive and defensive rallies, where blocking acts as the first defensive barrier and one of the few actions that can directly terminate rallies to score points. Match-analytics studies show that block-related indicators help discriminate winning from losing performances [1,2]. These indicators include block errors, block points, and suppression of opponent attacking efficiency [2].

Elite matches impose substantial cumulative load. Observational evidence from official competition reports more than one thousand jump events within match exposure [3,4]. Players perform repeated take-off and landing actions. These actions create repeated eccentric and concentric stress on the lower limbs [4,5]. This demand commonly leads to neuromuscular fatigue. Fatigue often reduces jump-related performance. Studies also show similar decrements after repeated block jump tasks [6].

Effective blocking requires more than just jumping high and athletes must rapidly detect cues, precisely coordinate timing, and maintain effective perception-action coupling. Fatigue impairs both physical force production and cognitive information-processing speed. These effects can undermine the combined height, speed, and timing demands of effective blocking [7,8].

Caffeine is a well-established ergogenic aid, with evidence supporting small but meaningful benefits across endurance-, strength-, power-, and team-sport tasks [9–13]. The International Society of Sports Nutrition position stand summarizes this evidence [10,11]. Meta-analysis and umbrella evidence also indicate that moderate caffeine doses produce small but consistent improvements in team-sport physical performance [11,12]. Whether these benefits transfer to volleyball actions that combine explosive output with rapid perception and movement timing under fatigue remains uncertain. Female and elite-athlete samples are comparatively limited [14,15], and individual responses may vary with metabolism, tolerability, and other athlete characteristics [14,16,17]. Current evidence indicates that habitual caffeine consumption does not consistently modify the acute ergogenic response, although individual variability remains relevant [18]. Dose-comparison studies under sport-specific fatigue are therefore warranted. Primary experimental work in female team-sport players has reported strength- and power-related responses to acute caffeine ingestion [15], whereas the systematic reviews, meta-analyzes, and umbrella review summarize evidence across studies [11,12,14].

Caffeinated chewing gum may be particularly feasible when the pre-performance window is short or when athletes wish to avoid additional fluid intake. Compared with gum, capsules, coffee, and caffeine-containing beverages generally require an earlier ingestion window to align caffeine availability with the intended performance period [19–21]. Gum also permits administration without added liquid and provides rapid buccal caffeine delivery, offering practical flexibility rather than representing an inherently superior administration route [19–24]. The present study did not compare caffeine-delivery methods. Primary pharmacokinetic research reported faster caffeine absorption from gum than from capsules [20]. A primary experimental study also quantified caffeine release and absorption from caffeinated gum matrices [21]. Team-sport studies suggest potential benefits for repeated high-intensity performance [25], but volleyball findings remain mixed across doses, tasks, and delivery forms [26–30]. Dose-specific effects of caffeinated gum on reactive blocking timing, block-jump mechanics, and related neuromuscular responses under fatigue therefore remain unclear [31].

Compared with prior volleyball-related caffeine studies that have more often focused on single-dose supplementation, general jump outcomes, or non-fatigued testing contexts [26–30], the present study combined three features in the same design: a volleyball-specific repeated blocking fatigue model, a direct comparison of two caffeine-gum doses, and the simultaneous assessment of reactive timing, jump mechanics, and exploratory neuromuscular indicators. This combination was intended to better reflect the practical demands of blocking performance under fatigue and to clarify whether different doses favor different functional components of performance. Therefore, this study compared placebo (PLA), 3 mg/kg caffeinated chewing gum (CAF3), and 6 mg/kg caffeinated chewing gum (CAF6) in elite female volleyball athletes using a volleyball-specific repeated blocking fatigue model. Assessments captured reactive blocking execution through block-jump mechanics, reactive timing-chain indices, general jump performance, and synchronized surface electromyography (sEMG). Because higher acute caffeine availability may better preserve neuromuscular activation and explosive force production, whereas a moderate dose may provide a more favorable arousal–control balance during time-constrained reactive tasks, a dose-specific pattern was expected. Accordingly, we hypothesized that caffeinated chewing gum would attenuate fatigue-induced decrements versus PLA, with CAF6 better preserving explosive output-oriented characteristics and CAF3 better supporting timing stability under reactive conditions. In this manuscript, timing stability is used as an interpretive description of limited change in the mean stationary and movement response times; no separate within-athlete or trial-to-trial variability metric was prespecified or analyzed.

## 2. Methods

### 2.1. Participants

An a priori power analysis was conducted in G*Power (version 3.1.9.7; Heinrich-Heine-Universität Düsseldorf, Düsseldorf, Germany) using a repeated-measures framework, with standardized Cohen’s f as the planning effect-size parameter [32]. Assuming a medium effect size (f = 0.25), a significance level of *α* = 0.05, statistical power of 1 − *β* = 0.80, and a within-subject correlation of r = 0.50 [33], the analysis indicated that at least 12 participants were required. Therefore, 15 elite female volleyball players were recruited to allow for potential attrition. All participants completed all experimental procedures. These effect-size and correlation values were planning assumptions rather than estimates from the present study.

Participants had a mean age of 21.5 ± 2.0 years, a height of 1.87 ± 0.07 m, a body mass of 69.5 ± 9.0 kg, a body mass index (BMI) of 22.57 ± 2.69 kg/m^2^, and a training experience of 7.3 ± 1.2 years. All participants held National First-Class Athlete certification in China and had competed in the National Games of China; these criteria operationalized elite status in the present study.

Eligibility criteria included: (i) active elite female volleyball players with ≥ 5 years of systematic training; (ii) a self-reported regular menstrual cycle during the prior 3 months with a typical cycle length of 28 ± 3 days and no oral contraceptive use [34]; (iii) low habitual caffeine intake (<2 mg/kg/day), classified using a food frequency questionnaire; and (iv) the ability to complete all visits and provide written informed consent. The questionnaire provided a categorical low-intake classification rather than a quantitative individual caffeine-intake estimate in mg/day or mg/kg/day. The sample was intentionally restricted to elite female volleyball players with low habitual caffeine intake to enhance sport-specific relevance while reducing variability in acute caffeine responsiveness. These sample characteristics should be considered when interpreting the generalizability of the findings. Exclusion criteria included: (i) lower-limb musculoskeletal injury within the prior 6 months or pain affecting jumping; (ii) known allergy to caffeine or gum ingredients; (iii) health risks incompatible with repeated high-intensity jumping; and (iv) non-compliance with pre-visit standardization requirements.

The study was approved by the Academic Ethics Committee of Capital University of Physical Education and Sports, Beijing, China, on 11 March 2026 (Approval No. 2026A075) and was conducted in accordance with the Declaration of Helsinki. Written informed consent was obtained from all participants, and withdrawal was permitted at any time without penalty.

### 2.2. Study design

A randomized, placebo-controlled crossover design was implemented using double-blinding procedures, with treatment codes concealed from participants and testing personnel until data collection was complete. Participants completed five laboratory visits: one familiarization visit, one baseline visit, and three formal experimental visits. The familiarization visit was used to familiarize participants with the environment, equipment, and procedures to reduce learning effects. The baseline visit recorded participant characteristics and collected individual reference values such as maximal reach height to monitor movement quality during the fatigue protocol.

The three formal experimental visits corresponded to PLA, CAF3, and CAF6. Each formal visit included two identical testing batteries: a post-supplementation, pre-fatigue time point (PRE) and an immediate post-fatigue time point (POST). Supplementation was followed by the standardized warm-up, PRE testing, the fatigue protocol, and immediate POST testing; PRE was therefore not a treatment-independent baseline. Condition order was assigned using a computer-generated random sequence, with efforts made to distribute treatment orders across participants to reduce sequence imbalance. A washout interval of at least 72 h and no more than 7 days was applied between formal visits, consistent with caffeine study guidance [10]. Given the acute administration protocol and the relatively short half-life of caffeine, pharmacological carryover was considered unlikely. The formal experimental visits were coded chronologically as Periods 1–3. The distribution of participants across the six treatment sequences is presented in Supplementary Table S1. To reduce circadian variation, all visits were scheduled within the same time window (09:00–11:00). Menstrual-cycle day was recorded at each visit, and formal testing sessions were scheduled within comparable menstrual-cycle phases whenever possible [34]. Across the sample, 11 of 15 participants completed all three formal trials within similar cycle phases, whereas 4 participants showed partial phase mismatch because of scheduling constraints. Menstrual-cycle information was recorded for descriptive control but was not included as a formal covariate in the primary statistical analyzes because of the limited sample size. Pre-visit state measures were collected to verify comparable readiness across visits (Section 2.4).

### 2.3. Supplementation and blinding protocol

Caffeine was administered as caffeinated chewing gum at nominal target doses of 3 and 6 mg/kg body mass. The quantity of caffeinated gum was calculated using each participant’s body mass and the manufacturer’s labeled caffeine content of 100 mg per whole piece; CAF3 and CAF6 therefore refer to nominal target-dose conditions. A dose-matching strategy was applied to maintain blinding and minimize condition identification by gum quantity. The number of gum pieces was matched to the maximum-dose condition. The remaining pieces were filled with placebo, keeping the total gum quantity identical across PLA, CAF3, and CAF6.

An independent staff member, who did not participate in the testing procedures, prepared the caffeinated and placebo gums. Stay Alert®, Military Energy Gum (Military Energy Gum, Marketright Inc., USA; mint flavor; 100 mg caffeine per piece) was used for caffeine conditions, and mint-flavored sugar-free gum was used as placebo. Original packaging was removed, and gums were repackaged into identical opaque packs with condition codes. The allocation key was held by this independent staff member until completion of data collection; participants and testing personnel were not informed of the assigned condition or nominal dose during the formal visits.

When required, whole gum pieces were divided into quarters to approximate the target dose in nominal 25-mg increments based on the manufacturer’s label. This fractionation approach has methodological precedent in a recent caffeine-gum crossover study that used 100-mg gum pellets cut into quarters [35]. Participants began chewing the coded gum about 15 min before the standardized warm-up and chewed for 10 min. Participants then expelled the gum residue and completed 5 min of quiet seated rest. The standardized warm-up followed supplementation; PRE testing was performed after the warm-up and before the fatigue protocol, and POST testing was performed immediately after the fatigue protocol. This timing was selected based on rapid buccal absorption from caffeinated gum and practical advantages compared with capsules [19,20].

### 2.4. Pre-trial standardization and state monitoring

Following standardized arrival procedures and a 5-min seated rest at each formal visit, staff assessed resting heart rate and blood pressure. Participants then completed the Profile of Mood States (POMS) [36] to document pre-trial mood state. The Pittsburgh Sleep Quality Index (PSQI) [37] was recorded descriptively to characterize sleep-related status across the study period rather than as an acute visit-level readiness measure. Because its 30-day recall windows overlapped between visits, the PSQI was not treated as an independent visit-level sleep or readiness measure.

### 2.5. Volleyball-specific fatigue protocol

A volleyball-specific repeated blocking protocol was implemented to induce substantial neuromuscular fatigue [6,38]. The protocol comprised 60 block jumps, organized as 20 sets of three consecutive jumps, and was performed near the net on an indoor court. Three target positions along the net were marked with cones corresponding to zones 4, 3, and 2.

A fixed within-set sequence (zone 4 to 3 to 2) was used to ensure consistency across visits. Participants used a shuffle or crossover step to reach each position, executed a two-foot vertical take-off with standardized blocking arm action, landed, and immediately moved to the next position. Between sets, participants walked to an 8-m recovery zone and returned to the starting position during an inter-set interval of approximately 10 s.

Movement quality was monitored using a target of 95% of each participant’s maximal reach height. Staff recorded whether each jump met the target. Staff provided immediate verbal feedback when jumps fell below the target. This criterion was used for monitoring and recording rather than early termination. Heart rate (HR) was recorded continuously during the protocol. Rating of perceived exertion (RPE) was recorded at the end of each set and immediately after protocol completion. RPE used a 0 to 10 scale [39]. Participants proceeded directly to POST testing after the final jump without additional recovery. Fatigue induction was evaluated not only by internal load markers (HR and RPE), but also by the immediate PRE-to-POST deterioration observed in jump-related mechanical outcomes and selected sEMG frequency-domain variables.

### 2.6. Performance testing procedures

During each formal visit, all performance tests were completed after supplementation and the standardized warm-up but before fatigue induction (PRE), and were repeated immediately after the fatigue protocol (POST). The test order was identical at both time points and included block jump (BJ), reactive blocking timing tasks, countermovement jump (CMJ), and drop jump (DJ). Three valid trials were collected for each test.

For each participant, condition, and time point, the arithmetic mean of three valid trials was used for BJ, CMJ, DJ, and reactive blocking timing outcomes. Kinetic and synchronized sEMG variables were extracted trial by trial and then averaged across the corresponding trials. Overall sequence time and its stationary and movement components were averaged across the same three timing trials.

For BJ, participants stood on a force plate in a standardized blocking-ready stance with feet parallel, performed a rapid countermovement, and executed a maximal two-foot vertical take-off with blocking arm action before landing and regaining balance on the force plate. Trials were classified as invalid if participants stepped off the plate, landed asymmetrically, performed a re-jump, or showed obvious instability; invalid trials were repeated immediately.

For reactive blocking timing, a SmartSpeed Pro light-based timing system (Fusion Sport, Australia) was synchronized with force-plate timestamps. Each trial consisted of two consecutive actions: (i) a stationary reactive block jump and (ii) a direction-cued movement reactive block jump. Stationary BJ response time was defined as the interval from onset of the first visual stimulus to take-off of the stationary block jump, and movement BJ response time was defined as the interval from onset of the directional stimulus to take-off of the movement block jump. Take-off was identified when vertical ground reaction force (vGRF) fell below 20 *N* for at least 20 ms. Overall sequence time was defined as the duration from onset of the first stimulus to take-off of the movement block jump, and it included the transition between the two actions.

For CMJ, participants performed CMJ on the force plate with hands on hips. Participants performed a rapid countermovement and then jumped maximally. Staff classified a trial as invalid if participants used arm swing or showed marked stepping.

For DJ, participants performed DJ from a 30 cm box with hands on hips. Participants stepped off the box without pushing upward. Participants landed with both feet and rebounded vertically. Participants aimed to minimize contact time and maximize jump height. Staff classified a trial as invalid if participants pushed off the box, landed on one foot, paused clearly, or performed a re-jump.

### 2.7. Instrumentation and signal processing

Vertical ground reaction force was collected using a force plate (Kistler 9287 C, Instrument Corp., Amherst, NY, USA) at 1000 Hz. Standard force-platform procedures were used to derive jump height and kinetic outputs [40,41], with jump height and power-related metrics extracted during the propulsive phase. CMJ modified reactive strength index was calculated as jump height divided by time to take-off and expressed in m/s.

For DJ, ground contact time was defined as the interval from initial contact to take-off. Initial contact was identified as vGRF > 20 N, and take-off was identified as vGRF < 20 N for at least 20 ms. Vertical stiffness during DJ was calculated as peak vGRF during ground contact divided by the maximum downward center-of-mass displacement during the same contact phase. Center-of-mass displacement was derived by integrating net vertical force to obtain velocity and displacement, where net vertical force was defined as vGRF minus body weight and normalized to body mass. DJ outcomes were calculated for each of the three valid trials and averaged within each participant, condition, and time point.

Surface EMG was recorded using a wireless system (Delsys Trigno, Inc., Natick, MA, USA) at 2000 Hz from eight muscles: gluteus maximus (GM), rectus femoris (RF), vastus medialis (VM), vastus lateralis (VL), biceps femoris (BF), semitendinosus (ST), medial gastrocnemius (MG), and tibialis anterior (TA). Skin preparation and electrode placement followed methodological recommendations [42]. Force-plate and sEMG signals were synchronized, and sEMG data were resampled to 1000 Hz to align with force-plate data. Signals were band-pass filtered (20–450 Hz), and a 50 Hz notch filter was applied when needed, followed by full-wave rectification and root mean square (RMS) smoothing. Muscle-specific MVC reference recordings were collected at each formal experimental visit before performance testing. The maximum valid MVC value for each muscle at that visit was used as the normalization reference. RMS and integrated EMG (iEMG) were normalized using that visit-specific reference, and the same denominator was applied to both PRE and POST.

sEMG outcomes were extracted within the propulsive phase, defined from the instant when vertical center-of-mass velocity changed from negative to positive until take-off (vGRF < 20 N for at least 20 ms). Extracted variables included mean RMS, iEMG, median frequency (MF), and muscle activation time (MAT). MF was estimated using Welch spectral estimation. MAT was determined using a 200 ms pre-movement baseline, with activation onset and offset identified using a threshold of baseline mean +3 standard deviations sustained for at least 25 ms [43]. For clarity and readability, the main text focuses on four functionally relevant muscles (GM, VM, RF, and BF), which are closely related to hip and knee extension and intersegmental timing control during blocking, whereas complete results for all eight recorded muscles are provided in Supplementary Tables S4–S7.

### 2.8. Blinding assessment and adverse effects

Immediately after each formal visit, participants independently completed a written standardized blinding-guess questionnaire without consulting investigators or other participants. Response options included caffeine, no caffeine, and uncertain/don’t know; participants who selected caffeine also reported the perceived dose. Forms were collected immediately, and responses from previous visits could not subsequently be revised. Adverse effects were assessed 24 h after each visit using a questionnaire covering insomnia or difficulty falling asleep, palpitations, tremor, gastrointestinal discomfort, and anxiety or nervousness, with space provided for additional comments. Incidence was summarized by condition. This assessment measured perceived caffeine presence and did not establish accurate identification of the exact 3- or 6-mg/kg condition.

### 2.9. Statistical analysis

Data were checked and cleaned in Microsoft Excel 2016 (Microsoft Corporation, Redmond, WA, USA) and analyzed using IBM SPSS Statistics version 26.0 (IBM Corp., Armonk, NY, USA). In line with the study aim, the primary outcomes were BJ peak power and reactive timing-chain indices (stationary response time, movement response time, and overall sequence time). Secondary outcomes included additional BJ mechanical variables and CMJ/DJ measures, whereas sEMG-derived variables were treated as exploratory mechanistic outcomes.

Repeated-measures ANOVA was used as the primary analytic framework to evaluate the main effects of Condition and Time, as well as the Condition × Time interaction, within the crossover repeated-measures design. Supplementary mixed-effects sensitivity analyzes of the four primary outcomes were performed to account for period and treatment sequence. Continuous variables are presented as mean ± SD. Normality was assessed using the Shapiro–Wilk test [44], and sphericity was evaluated using Mauchly’s test [45], with Greenhouse–Geisser correction applied when required [46]. Each participant contributed one trial-averaged value per condition and time point.

Pre-trial status across visits was compared using one-way repeated-measures ANOVA for resting heart rate, systolic blood pressure, diastolic blood pressure, and POMS score. Fatigue-related changes in the study outcomes were analyzed using two-way repeated-measures ANOVA. When significant omnibus effects or interactions were detected, Bonferroni-adjusted post hoc comparisons were performed within each outcome. For significant Time × Condition interactions, simple effects were examined by comparing PRE versus POST within each condition and by comparing the three condition contrasts at POST; post-hoc pairwise testing was not performed for outcomes without a significant interaction. Statistical significance was set at *p* < 0.05, and effect sizes were reported as partial eta squared (ηp^2^). Adverse effects and blinding responses were summarized descriptively as frequencies and percentages, and no inferential statistical comparisons were performed for these variables.

For descriptive interpretation, percentage change was calculated from condition-specific group means as 100 × (POST − PRE)/PRE. No outcome-specific threshold for a smallest worthwhile change, minimal detectable change, or practical equivalence was prespecified. Accordingly, little change denotes a small displayed group-mean difference only and was not interpreted as equivalence or absence of change.

Supplementary mixed-effects sensitivity analyzes of the four primary outcomes were performed to account for period and treatment sequence. Fixed effects included condition, time, condition × time, period, period × time, and treatment sequence, with random intercepts for participants and visits within participants. Models were fitted using restricted maximum likelihood, with Kenward–Roger inference for fixed effects and contrasts. Adjusted between-condition differences at POST and in PRE-to-POST change were estimated, with change defined as POST minus PRE. Within each outcome and estimand, Bonferroni adjustment was applied to the *p*-values and 95% confidence intervals for the three condition comparisons. Supplementary PRE condition comparisons used participant-level three-trial means in repeated-measures ANOVA with Greenhouse–Geisser correction, implemented in Python 3.12.14.

## 3. Results

### 3.1. Data completeness and pre-trial status

All participants completed all three experimental conditions and the prescribed 60-jump protocol without early termination, and complete outcome records were available for the analyzes. Table 1 presents the pre-trial physiological and mood-state measures; no statistically significant condition differences were detected for HR, SBP, DBP, or POMS (all *p* ≥ 0.224). These results indicate similar pre-trial readiness measures across visits; they do not establish equivalence of the post-supplementation PRE performance outcomes. Across outcomes, the transition from PRE to POST was accompanied by broad decrements in jump-related performance and selected frequency-domain sEMG indices, supporting successful fatigue induction by the volleyball-specific repeated blocking protocol.

**Table 1.** Pre-trial physiological measures and mood-state scores across the experimental conditions.

Variable	PLA	CAF3	CAF6	F	*p*
Resting heart rate (HR, bpm)	59 ± 12	59 ± 11	61 ± 10	0.47	0.629
Systolic blood pressure (SBP, mmHg)	114 ± 6	115 ± 6	117 ± 5	1.18	0.321
Diastolic blood pressure (DBP, mmHg)	67 ± 8	69 ± 8	68 ± 7	1.57	0.224
Profile of Mood States (POMS, score)	108 ± 2	107 ± 2	109 ± 1	0.94	0.245

Note: Data are presented as mean ± SD. PLA = placebo; CAF3 = 3 mg/kg caffeinated chewing gum; CAF6 = 6 mg/kg caffeinated chewing gum. Pre-trial differences across conditions were assessed using a one-way repeated-measures ANOVA.

### 3.2. Primary outcomes

#### 3.2.1. BJ peak power

Table 2A presents the BJ peak power results before and after the fatigue protocol. BJ peak power showed a significant Time × Condition interaction, F = 4.65, *p* = 0.018, ηp^2^ = 0.249. Peak power decreased from PRE to POST in all three conditions, but the magnitude of reduction differed across conditions. The decline was greatest in PLA at 13.9%, followed by CAF3 at 11.7%, whereas CAF6 showed the smallest reduction at 6.9%. Post-hoc comparisons at POST showed significant differences between CAF6 and PLA, *p* = 0.005, and between CAF6 and CAF3, *p* < 0.001, whereas CAF3 did not differ significantly from PLA, *p* = 0.072.

**Table 2A.** Block jump (BJ) peak power before (PRE) and after (POST) the fatigue protocol.

Variable	Condition	PRE	POST	Time × Condition F	*p*	ηp^2^	Post-hoc PLA vs CAF3	PLA vs CAF6	CAF3 vs CAF6
BJ peak power (W)	PLA	3429 ± 512	2953 ± 615	4.65	0.018	0.249	0.072	0.005	<0.001
	CAF3	3445 ± 548	3041 ± 507						
	CAF6	3405 ± 803	3171 ± 494						

Note: Data are presented as mean ± SD. PLA = placebo; CAF3 = 3 mg/kg caffeinated chewing gum; CAF6 = 6 mg/kg caffeinated chewing gum. PRE = post-supplementation, pre-fatigue; POST = post-fatigue. Post-hoc *p*-values reflect pairwise comparisons at POST, performed only when a significant Time × Condition interaction was observed. These results are from the original repeated-measures ANOVA; analyzes accounting for period and treatment sequence are reported in Supplementary Tables S2 and S3.

#### 3.2.2. Reactive timing-chain characteristics

The reactive timing-chain outcomes are summarized in Table 2B. Stationary BJ response time showed a significant Time × Condition interaction, F = 25.43, *p <* 0.001, ηp^2^ = 0.645. Movement BJ response time also showed a significant interaction, F = 33.44, *p <* 0.001, ηp^2^ = 0.705. Overall sequence time likewise showed a significant interaction, F = 27.57, *p <* 0.001, ηp^2^ = 0.663.

**Table 2B.** Reactive blocking timing characteristics before (PRE) and after (POST) the fatigue protocol.

Variable	Condition	PRE	POST	Time × Condition F	*p*	ηp^2^	Post-hoc PLA vs CAF3	PLA vs CAF6	CAF3 vs CAF6
Stationary BJ response time (s)	PLA	0.89 ± 0.21	1.06 ± 0.27	25.43	<0.001	0.645	0.006	0.003	0.006
	CAF3	1.01 ± 0.26	1.01 ± 0.24						
	CAF6	1.06 ± 0.29	0.96 ± 0.21						
Movement BJ response time (s)	PLA	2.71 ± 0.18	3.28 ± 0.66	33.44	<0.001	0.705	0.006	0.004	0.028
	CAF3	2.97 ± 0.26	2.97 ± 0.34						
	CAF6	2.95 ± 0.29	3.08 ± 0.46						
Overall sequence time (s)	PLA	6.40 ± 0.73	7.58 ± 1.53	27.57	<0.001	0.663	0.008	0.004	0.022
	CAF3	6.71 ± 0.75	7.14 ± 1.07						
	CAF6	6.75 ± 0.76	6.95 ± 0.99						

Note: Data are presented as mean ± SD. PLA = placebo; CAF3 = 3 mg/kg caffeinated chewing gum; CAF6 = 6 mg/kg caffeinated chewing gum. PRE = post-supplementation, pre-fatigue; POST = post-fatigue. Post-hoc *p*-values reflect pairwise comparisons at POST following a significant Time × Condition interaction. These results are from the original repeated-measures ANOVA; analyzes accounting for period and treatment sequence are reported in Supplementary Tables S2 and S3.

Because PRE was collected after supplementation and before fatigue, it was a post-supplementation measurement rather than an untreated baseline. Descriptive PRE means differed across conditions for stationary response time (PLA 0.89 ± 0.21 s, CAF3 1.01 ± 0.26 s, CAF6 1.06 ± 0.29 s), movement response time (PLA 2.71 ± 0.18 s, CAF3 2.97 ± 0.26 s, CAF6 2.95 ± 0.29 s), and overall sequence time (PLA 6.40 ± 0.73 s, CAF3 6.71 ± 0.75 s, CAF6 6.75 ± 0.76 s). These descriptive PRE differences were considered together with POST values and PRE-to-POST changes when interpreting the condition effects. Supplementary repeated-measures comparisons of PRE values showed condition differences in stationary response time, movement response time, and overall sequence time (all Greenhouse–Geisser-corrected *p* < 0.001), whereas the PRE comparison for BJ peak power was not significant (*p* = 0.853; Supplementary Table S2, Panel B).

For stationary response time, the group mean increased by 19.1% in PLA and decreased by 9.4% in CAF6. In CAF3, the displayed group mean was 1.01 s at both PRE and POST, indicating little change at the reported precision. Post-hoc comparisons at POST showed significant differences between PLA and CAF3, *p* = 0.006, between PLA and CAF6, *p* = 0.003, and between CAF3 and CAF6, *p* = 0.006.

For movement response time, the group mean increased by 21.0% in PLA and by 4.4% in CAF6. In CAF3, the displayed group mean was 2.97 s at both PRE and POST, indicating little change at the reported precision. Post-hoc comparisons at POST showed significant differences between PLA and CAF3, *p* = 0.006, between PLA and CAF6, *p* = 0.004, and between CAF3 and CAF6, *p* = 0.028.

For overall sequence time, PLA increased by 18.4%, CAF3 by 6.4%, and CAF6 by 3.0%; CAF6 therefore showed the smallest descriptive deterioration in this integrated timing outcome. Post-hoc comparisons at POST showed significant differences between PLA and CAF3, *p* = 0.008, between PLA and CAF6, *p* = 0.004, and between CAF3 and CAF6, *p* = 0.022.

**Figure 1.**
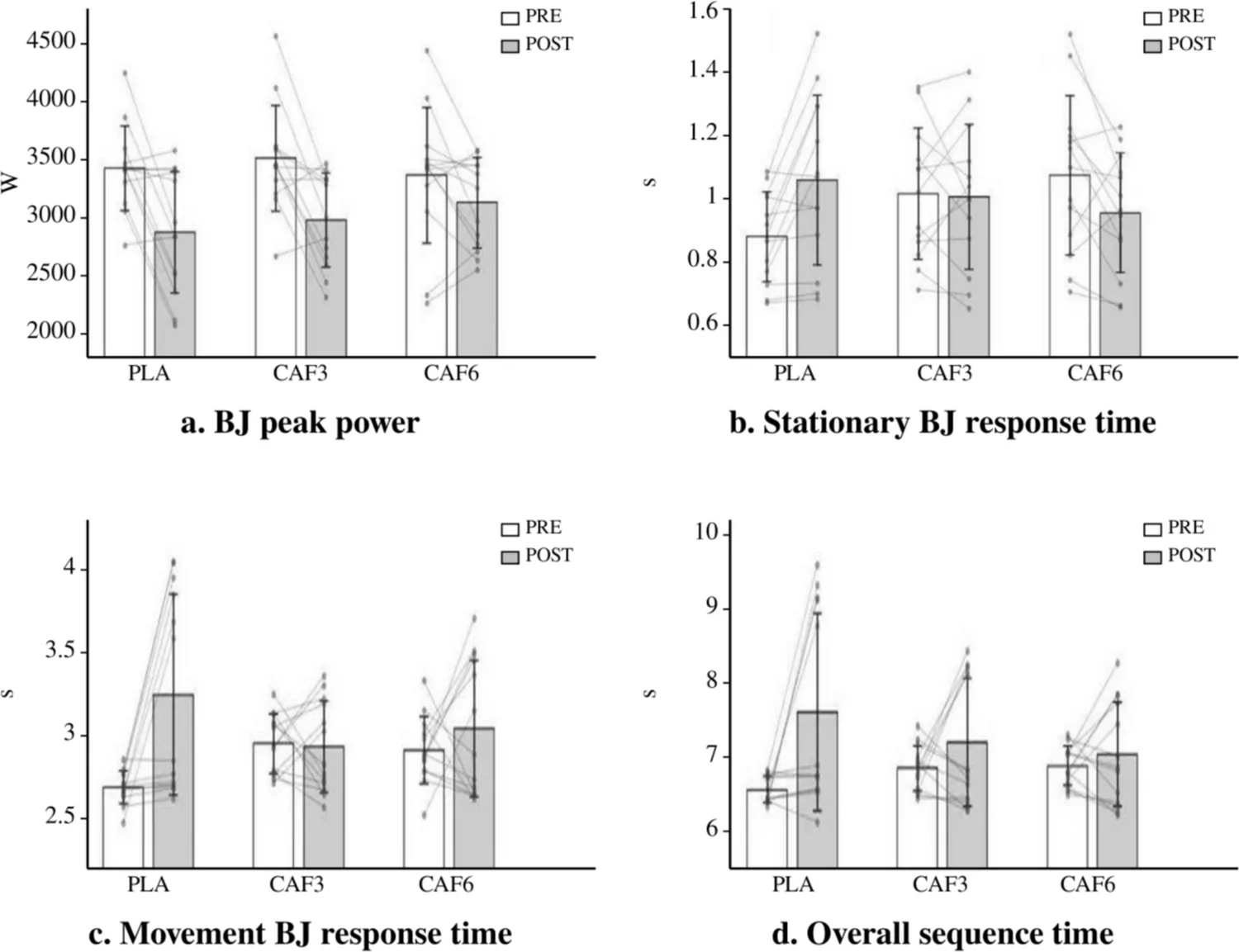
Individual response patterns for the primary outcomes across placebo (PLA), 3 mg/kg caffeinated chewing gum (CAF3), and 6 mg/kg caffeinated chewing gum (CAF6). Panels show (a) BJ peak power, (b) stationary BJ response time, (c) movement BJ response time, and (d) overall sequence time at PRE and POST. Bars represent group means, error bars indicate SD, and lines connect repeated measures from the same participant.

Figure 1 provides a descriptive visualization of individual response patterns; the timing panels were not analyzed as a separate test of timing stability.

#### 3.2.3. Period and treatment sequence sensitivity analysis

After adjustment for period and treatment sequence, condition × time interactions were statistically significant for BJ peak power, stationary BJ response time, movement BJ response time, and overall sequence time (all *p* ≤ 0.022). No statistical evidence of period, period × time, or treatment sequence effects was detected for these outcomes (all *p* ≥ 0.540; Supplementary Table S2, Panel A). Adjusted condition contrasts are presented in Supplementary Table S3. Adjusted POST differences between CAF6 and CAF3 were not significant for BJ peak power (*p* = 0.112), stationary response time (*p* = 0.459), or movement response time (*p* = 0.116), whereas overall sequence time differed (*p* = 0.012).

### 3.3. Secondary outcomes

Table 3 summarizes the additional BJ mechanical outcomes together with the CMJ and DJ outcomes. BJ jump height showed no Time × Condition interaction, *p* = 0.352, ηp^2^ = 0.072, and decreased from PRE to POST in all conditions with similar reductions in PLA at 14.7%, CAF3 at 14.3%, and CAF6 at 14.3%. BJ take-off time also showed no Time × Condition interaction, *p* = 0.524, ηp^2^ = 0.045. Take-off time increased in PLA by 19.7%, whereas the change was minimal in CAF3 at 1.3% and in CAF6 at 5.1% (Figure 2).

**Table 3.** Additional BJ Mechanical Outcomes, CMJ and DJ performance before (PRE) and after (POST) the fatigue protocol.

Variable	Condition	PRE	POST	Time × Condition F	*p*	ηp^2^	Post-hoc PLA vs CAF3	PLA vs CAF6	CAF3 vs CAF6
BJ jump height (m)	PLA	0.34 ± 0.03	0.29 ± 0.06	1.08	0.352	0.072	−	−	−
	CAF3	0.35 ± 0.05	0.30 ± 0.05						
	CAF6	0.35 ± 0.04	0.30 ± 0.04						
BJ take-off time (s)	PLA	0.71 ± 0.22	0.85 ± 0.30	0.66	0.524	0.045	−	−	−
	CAF3	0.79 ± 0.27	0.80 ± 0.27						
	CAF6	0.78 ± 0.26	0.82 ± 0.15						
CMJ jump height (m)	PLA	0.30 ± 0.03	0.26 ± 0.05	0.06	0.945	0.004	−	−	−
	CAF3	0.31 ± 0.03	0.27 ± 0.04						
	CAF6	0.32 ± 0.04	0.27 ± 0.04						
CMJ mRSI (m/s)	PLA	0.41 ± 0.07	0.50 ± 0.10	22.25	<0.001	0.614	0.004	0.003	0.011
	CAF3	0.47 ± 0.08	0.47 ± 0.08						
	CAF6	0.48 ± 0.09	0.45 ± 0.07						
CMJ mean propulsive power (W)	PLA	1679 ± 459	1437 ± 500	7.19	0.003	0.339	0.003	0.025	0.163
	CAF3	1672 ± 496	1499 ± 483						
	CAF6	1626 ± 505	1535 ± 445						
DJ jump height (m)	PLA	0.30 ± 0.03	0.26 ± 0.05	5.36	0.011	0.277	0.005	0.005	0.019
	CAF3	0.30 ± 0.04	0.27 ± 0.05						
	CAF6	0.30 ± 0.03	0.28 ± 0.04						
DJ contact time (s)	PLA	0.45 ± 0.07	0.54 ± 0.15	26.61	<0.001	0.655	0.010	0.011	0.036
	CAF3	0.51 ± 0.10	0.49 ± 0.09						
	CAF6	0.52 ± 0.11	0.51 ± 0.11						
DJ vertical stiffness (N·m^−1^)	PLA	2695 ± 604	2384 ± 782	4.95	0.014	0.261	0.076	0.009	0.003
	CAF3	2718 ± 789	2432 ± 732						
	CAF6	2486 ± 596	2506 ± 691						

Note: Data are presented as mean ± SD. PLA = placebo; CAF3 = 3 mg/kg caffeinated chewing gum; CAF6 = 6 mg/kg caffeinated chewing gum. PRE = post-supplementation, pre-fatigue; POST = post-fatigue. Post-hoc *p*-values reflect pairwise comparisons at POST, performed only when a significant Time × Condition interaction was observed. Dashes (−) indicate that post-hoc testing was not applicable.

**Figure 2.**
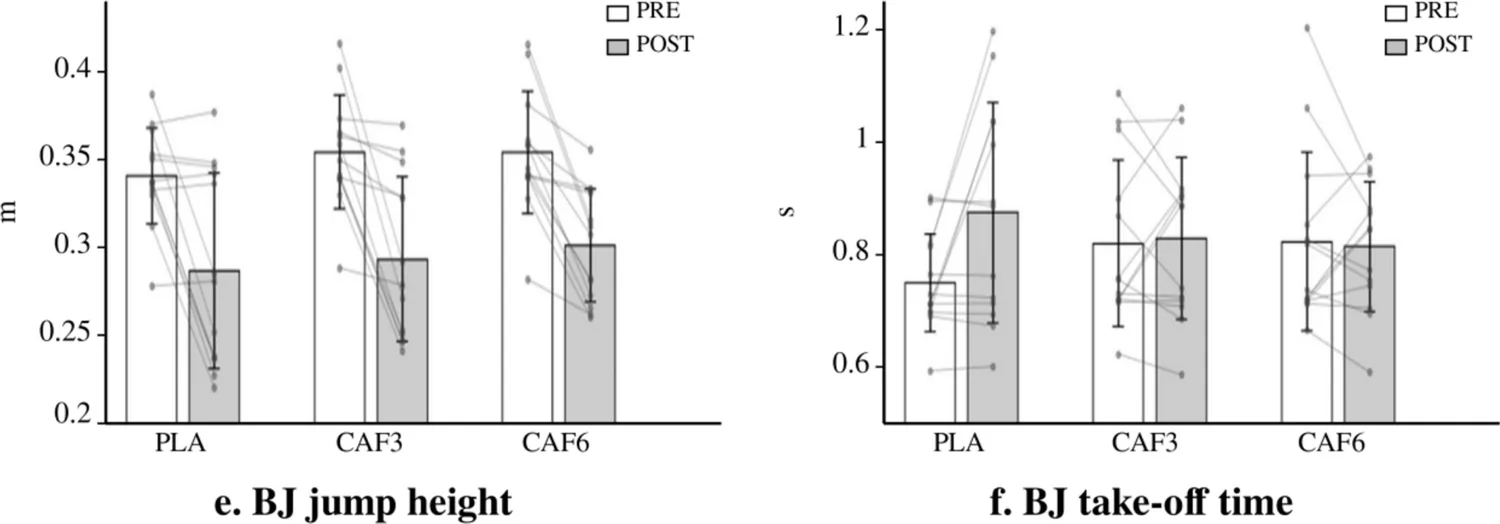
Descriptive individual response patterns for additional block-jump mechanical outcomes across placebo (PLA), 3 mg/kg caffeinated chewing gum (CAF3), and 6 mg/kg caffeinated chewing gum (CAF6). Panels show (e) BJ jump height and (f) BJ take-off time at PRE and POST. Bars represent group means, error bars indicate SD, and lines connect repeated measures from the same participant. No significant Condition × Time interaction was observed for either outcome; accordingly, the figure is presented for descriptive completeness.

CMJ jump height is shown in Figure 3 for descriptive completeness, but no Time × Condition interaction was observed, *p* = 0.945, ηp^2^ = 0.004, and the variable decreased from PRE to POST in all conditions, with reductions of 13.3% in PLA, 12.9% in CAF3, and 15.6% in CAF6. CMJ modified reactive strength index showed a significant Time × Condition interaction, F = 22.25, *p* < 0.001, ηp^2^ = 0.614. However, the direction of change did not support a caffeine-related attenuation of fatigue-induced decline. CMJ mRSI increased in PLA from 0.41 ± 0.07 to 0.50 ± 0.10 m/s, showed little change in the CAF3 group mean from 0.47 ± 0.08 to 0.47 ± 0.08 m/s, and decreased slightly in CAF6 from 0.48 ± 0.09 to 0.45 ± 0.07 m/s. Therefore, this outcome was treated as a divergent ratio-derived response and was not interpreted as evidence that caffeine preserved CMJ reactive strength under fatigue. Post-hoc comparisons at POST showed significant differences between PLA and CAF3, *p* = 0.004, between PLA and CAF6, *p* = 0.003, and between CAF3 and CAF6, *p* = 0.011. CMJ mean propulsive power showed a significant Time × Condition interaction, F = 7.19, *p* = 0.003, ηp^2^ = 0.339. The decline was greatest in PLA and smaller in both caffeine conditions. Post-hoc comparisons at POST showed significant differences between PLA and CAF3, *p* = 0.003, and between PLA and CAF6, *p* = 0.025, whereas CAF3 did not differ significantly from CAF6, *p* = 0.163.

DJ contact time showed a significant Time × Condition interaction, F = 26.61, *p* < 0.001, ηp^2^ = 0.655. Contact time increased in PLA by 20.0%, whereas it decreased slightly in CAF3 by 3.9% and in CAF6 by 1.9%. Post-hoc comparisons at POST showed significant differences between PLA and CAF3, *p* = 0.010, between PLA and CAF6, *p* = 0.011, and between CAF3 and CAF6, *p* = 0.036. DJ jump height also showed a significant Time × Condition interaction, F = 5.36, *p* = 0.011, ηp^2^ = 0.277. Jump height decreased in all conditions, but the reduction was greatest in PLA at 13.3%, followed by CAF3 at 10.0%, whereas CAF6 showed the smallest decline at 6.7%. Post-hoc comparisons at POST showed significant differences between PLA and CAF3, *p* = 0.005, between PLA and CAF6, *p* = 0.005, and between CAF3 and CAF6, *p* = 0.019. DJ vertical stiffness showed a significant Time × Condition interaction, F = 4.95, *p* = 0.014, ηp^2^ = 0.261. Vertical stiffness decreased in PLA by 11.5% and in CAF3 by 10.5%, but was largely maintained in CAF6, with a change of 0.8%. Post-hoc comparisons at POST showed that CAF6 was significantly higher than PLA, *p* = 0.009, and higher than CAF3, *p* = 0.003, whereas PLA did not differ significantly from CAF3, *p* = 0.076.

**Figure 3.**
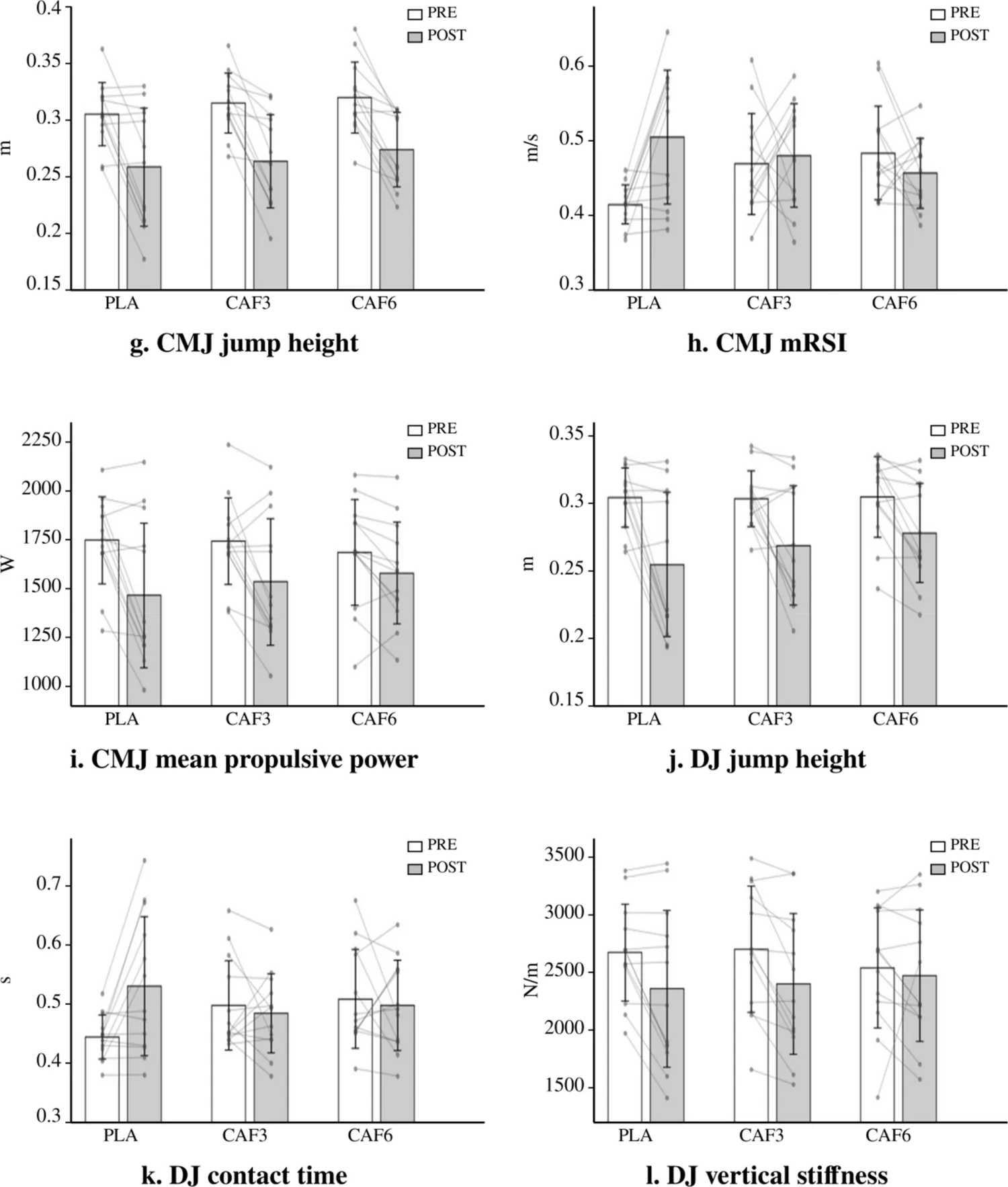
Individual response patterns for selected CMJ and DJ outcomes across placebo (PLA), 3 mg/kg caffeinated chewing gum (CAF3), and 6 mg/kg caffeinated chewing gum (CAF6). Panels show (g) CMJ jump height, (h) CMJ mRSI, (i) CMJ mean propulsive power, (j) DJ jump height, (k) DJ contact time, and (l) DJ vertical stiffness at PRE and POST. Bars represent group means, error bars indicate SD, and lines connect repeated measures from the same participant. CMJ jump height is included for descriptive completeness despite a nonsignificant interaction, whereas the remaining panels correspond to outcomes with significant Condition × Time interactions reported in Table 3.

Figure 3 should be interpreted with greatest emphasis on CMJ mean propulsive power, DJ jump height, DJ contact time, and DJ vertical stiffness, whereas CMJ jump height is included for descriptive completeness. Although CMJ mRSI showed a significant Condition × Time interaction, its direction differed from the hypothesized caffeine-related fatigue-protection pattern. Specifically, CMJ mRSI increased in PLA, showed little change in the CAF3 group mean, and decreased slightly in CAF6 from PRE to POST. Because CMJ mRSI is a ratio-derived index based on jump height and time to take-off, the time component may have contributed to this divergent pattern; however, CMJ time to take-off was not analyzed as a separate outcome, so this interpretation remains tentative. Therefore, CMJ mRSI was interpreted cautiously and was not used as evidence of caffeine-mediated preservation of reactive strength.

### 3.4. Exploratory neuromuscular outcomes

The main text focuses on four functionally relevant muscles (GM, VM, RF, and BF), whereas complete sEMG results for all eight recorded muscles are provided in Supplementary Tables S4–S7. These muscles were emphasized because they are directly involved in hip and knee extension and in the timing demands of blocking take-off.

#### 3.4.1. Integrated EMG (iEMG)

Integrated EMG showed significant Time × Condition interactions in all four muscles. GM showed F = 26.10, *p <* 0.001, ηp^2^ = 0.667. RF showed F = 19.77, *p <* 0.001, ηp^2^ = 0.603. VM showed F = 318.05, *p <* 0.001, ηp^2^ = 0.961. BF showed F = 42.93, *p <* 0.001, ηp^2^ = 0.768. Post-hoc comparisons at POST showed that CAF6 differed from PLA for GM, *p =* 0.025, for RF, *p =* 0.027, for VM, *p =* 0.020, and for BF, *p <* 0.001. CAF6 also differed from CAF3 for GM, *p =* 0.049, and for BF, *p =* 0.002. CAF3 did not differ from PLA for these muscles, GM *p =* 0.549, RF *p =* 0.104, VM *p =* 0.710, and BF *p =* 0.111. Table 4 reports these results.

**Table 4.** Block jump integrated electromyography (iEMG) of primary muscles before (PRE) and after (POST) the fatigue protocol.

Variable	Condition	PRE	POST	Time × Condition F	*p*	ηp^2^	Post-hoc PLA vs CAF3	PLA vs CAF6	CAF3 vs CAF6
GM	PLA	1.43 ± 1.65	1.52 ± 1.77	26.10	<0.001	0.667	0.549	0.025	0.049
	CAF3	1.81 ± 2.09	1.56 ± 1.89						
	CAF6	1.17 ± 1.21	1.24 ± 1.39						
RF	PLA	0.76 ± 0.25	0.72 ± 0.23	19.77	<0.001	0.603	0.104	0.027	0.326
	CAF3	0.82 ± 0.37	0.77 ± 0.30						
	CAF6	0.82 ± 0.33	0.80 ± 0.26						
VM	PLA	0.85 ± 0.24	0.72 ± 0.19	318.05	<0.001	0.961	0.710	0.020	0.215
	CAF3	0.84 ± 0.23	0.75 ± 0.17						
	CAF6	0.85 ± 0.36	0.85 ± 0.31						
BF	PLA	0.36 ± 0.11	0.39 ± 0.11	42.93	<0.001	0.768	0.111	<0.001	0.002
	CAF3	0.35 ± 0.13	0.36 ± 0.11						
	CAF6	0.35 ± 0.14	0.31 ± 0.11						

Note: Data are presented as mean ± SD in normalized arbitrary units (a.u.). For each muscle, normalization used the maximum valid MVC reference obtained at the same experimental visit before performance testing; the same reference was applied to PRE and POST. PLA = placebo; CAF3 = 3 mg/kg caffeinated chewing gum; CAF6 = 6 mg/kg caffeinated chewing gum. PRE = post-supplementation, pre-fatigue; POST = post-fatigue. Post-hoc *p*-values reflect pairwise comparisons at POST following a significant Time × Condition interaction.

#### 3.4.2. Root mean square (RMS) amplitude

RMS amplitude showed significant Time × Condition interactions for RF, F = 25.72, *p <* 0.001, ηp^2^ = 0.664, and for VM, F = 67.32, *p <* 0.001, ηp^2^ = 0.838. GM showed no interaction, *p =* 0.776, ηp^2^ = 0.019. BF also showed no interaction, *p =* 0.996, ηp^2^ < 0.001. Post-hoc comparisons at POST showed that VM RMS differed between CAF6 and PLA, *p =* 0.001, and between CAF6 and CAF3, *p =* 0.027. CAF3 did not differ from PLA for VM RMS, *p =* 0.074. RF RMS showed no between-condition differences at POST, all *p* ≥ 0.107. Table 5 reports these results.

**Table 5.** Block jump root mean square (RMS) amplitude of primary muscles before (PRE) and after (POST) the fatigue protocol.

Variable	Condition	PRE	POST	Time × Condition F	*p*	ηp^2^	Post-hoc PLA vs CAF3	PLA vs CAF6	CAF3 vs CAF6
GM	PLA	0.78 ± 0.38	0.83 ± 0.42	0.26	0.776	0.019	−	−	−
	CAF3	0.92 ± 1.00	0.91 ± 0.39						
	CAF6	0.87 ± 1.11	0.82 ± 0.46						
RF	PLA	1.04 ± 0.27	0.97 ± 0.23	25.72	<0.001	0.664	0.107	0.687	0.167
	CAF3	1.12 ± 0.60	1.08 ± 0.41						
	CAF6	0.99 ± 0.47	0.94 ± 0.41						
VM	PLA	1.26 ± 0.35	1.07 ± 0.30	67.32	<0.001	0.838	0.074	0.001	0.027
	CAF3	1.32 ± 0.65	1.21 ± 0.46						
	CAF6	1.39 ± 0.65	1.45 ± 0.50						
BF	PLA	0.58 ± 0.22	0.62 ± 0.24	0.00	0.996	<0.001	−	−	−
	CAF3	0.62 ± 0.62	0.64 ± 0.30						
	CAF6	0.68 ± 1.00	0.59 ± 0.31						

Note. Data are presented as mean ± SD in normalized arbitrary units (a.u.). For each muscle, normalization used the maximum valid MVC reference obtained at the same experimental visit before performance testing; the same reference was applied to PRE and POST. PLA = placebo; CAF3 = 3 mg/kg caffeinated chewing gum; CAF6 = 6 mg/kg caffeinated chewing gum. PRE = post-supplementation, pre-fatigue; POST = post-fatigue. Post-hoc *p*-values reflect pairwise comparisons at POST, performed only when a significant Time × Condition interaction was observed. Dashes (−) indicate that post-hoc testing was not applicable.

#### 3.4.3. Median frequency (MF)

Median frequency showed significant Time × Condition interactions in all four muscles. GM showed F = 214.20, *p <* 0.001, ηp^2^ = 0.943. RF showed F = 196.08, *p <* 0.001, ηp^2^ = 0.938. VM showed F = 90.37, *p <* 0.001, ηp^2^ = 0.874. BF showed F = 183.11, *p <* 0.001, ηp^2^ = 0.934. Post-hoc comparisons at POST showed muscle-specific patterns. GM differed between CAF6 and PLA, *p =* 0.002, and between CAF6 and CAF3, *p =* 0.026. GM did not differ between PLA and CAF3, *p =* 0.493. RF differed between PLA and CAF3, *p <* 0.001, and between PLA and CAF6, *p =* 0.001. RF did not differ between CAF3 and CAF6, *p =* 0.879. VM differed between PLA and CAF3, *p =* 0.008, and between PLA and CAF6, *p =* 0.017. VM did not differ between CAF3 and CAF6, *p =* 0.537. BF differed between PLA and CAF3, *p =* 0.002, while BF did not differ between PLA and CAF6, *p =* 0.118. Table 6 reports these results.

**Table 6.** Block jump median frequency (MF) of primary muscles before (PRE) and after (POST) the fatigue protocol.

Variable	Condition	PRE	POST	Time × Condition F	*p*	ηp^2^	Post-hoc PLA vs CAF3	PLA vs CAF6	CAF3 vs CAF6
GM	PLA	85.8 ± 35.2	77.3 ± 31.3	214.20	<0.001	0.943	0.493	0.002	0.026
	CAF3	83.9 ± 34.7	78.7 ± 27.9						
	CAF6	85.3 ± 39.6	89.4 ± 40.4						
RF	PLA	88.6 ± 12.9	79.8 ± 11.1	196.08	<0.001	0.938	<0.001	0.001	0.879
	CAF3	91.4 ± 20.1	86.4 ± 12.6						
	CAF6	86.6 ± 16.6	86.4 ± 10.4						
VM	PLA	82.0 ± 10.4	73.8 ± 7.8	90.37	<0.001	0.874	0.008	0.017	0.537
	CAF3	82.8 ± 17.6	79.1 ± 11.5						
	CAF6	83.3 ± 19.2	81.7 ± 14.4						
BF	PLA	85.2 ± 15.1	76.9 ± 13.1	183.11	<0.001	0.934	0.002	0.118	0.891
	CAF3	85.6 ± 21.1	81.7 ± 11.9						
	CAF6	80.8 ± 18.8	82.0 ± 17.2						

Note: Data are presented as mean ± SD in hertz (Hz). PLA = placebo; CAF3 = 3 mg/kg caffeinated chewing gum; CAF6 = 6 mg/kg caffeinated chewing gum. PRE = post-supplementation, pre-fatigue; POST = post-fatigue. Post-hoc *p*-values reflect pairwise comparisons at POST following a significant Time × Condition interaction.

#### 3.4.4. Muscle activation time (MAT)

Muscle activation time showed significant Time × Condition interactions for GM, F = 18.63, *p <* 0.001, ηp^2^ = 0.589, for RF, F = 4.10, *p =* 0.028, ηp^2^ = 0.240, for VM, F = 4.42, *p =* 0.022, ηp^2^ = 0.254, and for BF, F = 4.36, *p =* 0.023, ηp^2^ = 0.251. Post-hoc comparisons at POST showed that CAF6 differed from PLA for GM, *p =* 0.048, for RF, *p =* 0.033, and for BF, *p =* 0.045. BF also differed between CAF3 and CAF6, *p =* 0.032. VM showed a significant interaction, but post-hoc comparisons did not show clear between-condition separation, all *p* > 0.05. Table 7 reports these results.

**Table 7.** Block jump muscle activation time (MAT) of primary muscles before (PRE) and after (POST) the fatigue protocol.

Variable	Condition	PRE	POST	Time × Condition F	*p*	ηp^2^	Post-hoc PLA vs CAF3	PLA vs CAF6	CAF3 vs CAF6
GM	PLA	0.19 ± 0.11	0.21 ± 0.10	18.63	<0.001	0.589	0.717	0.048	0.186
	CAF3	0.17 ± 0.20	0.18 ± 0.15						
	CAF6	0.16 ± 0.19	0.14 ± 0.15						
RF	PLA	0.15 ± 0.10	0.17 ± 0.10	4.10	0.028	0.240	0.748	0.033	0.066
	CAF3	0.18 ± 0.19	0.18 ± 0.16						
	CAF6	0.14 ± 0.17	0.12 ± 0.12						
VM	PLA	0.12 ± 0.07	0.13 ± 0.07	4.42	0.022	0.254	0.953	0.384	0.518
	CAF3	0.12 ± 0.18	0.13 ± 0.13						
	CAF6	0.16 ± 0.19	0.15 ± 0.12						
BF	PLA	0.22 ± 0.13	0.24 ± 0.16	4.36	0.023	0.251	0.604	0.045	0.032
	CAF3	0.23 ± 0.24	0.24 ± 0.17						
	CAF6	0.17 ± 0.23	0.13 ± 0.14						

Note: Data are presented as mean ± SD in seconds (s). PLA = placebo; CAF3 = 3 mg/kg caffeinated chewing gum; CAF6 = 6 mg/kg caffeinated chewing gum. PRE = post-supplementation, pre-fatigue; POST = post-fatigue. Post-hoc *p*-values reflect pairwise comparisons at POST following a significant Time × Condition interaction.

### 3.5. Adverse effects and blinding integrity

Table 8 summarizes adverse effects and blinding assessments across conditions. Adverse effects were self-reported 24 h after each trial and showed a dose-related pattern, with the highest frequency observed in CAF6, fewer events in CAF3, and minimal complaints in PLA. The most commonly reported symptom was increased urination, followed by gastrointestinal discomfort and sleep difficulty/insomnia. No participants reported tremor, anxiety/nervousness, or other symptoms in any condition.

**Table 8.** Self-reported adverse effects and blinding assessments across experimental conditions.

Panel A. Blinding guess			
Item	PLA	CAF3	CAF6
Judged Caffeine ingested	0 (0.0)	8 (53.3)	12 (80.0)
Judged No caffeine ingested	13 (86.7)	3 (20.0)	2 (13.3)
Judged Uncertain	2 (13.3)	4 (26.7)	1 (6.7)
**Panel B. Adverse effects**			
Item	PLA	CAF3	CAF6
Sleep difficulty/insomnia	0 (0.0)	1 (6.7)	2 (13.3)
Increased urination	1 (6.7)	4 (26.7)	6 (40.0)
Heart palpitations	0 (0.0)	0 (0.0)	1 (6.7)
Tremor	0 (0.0)	0 (0.0)	0 (0.0)
Gastrointestinal discomfort	1 (6.7)	0 (0.0)	3 (20.0)
Anxiety/nervousness	0 (0.0)	0 (0.0)	0 (0.0)
Other	0 (0.0)	0 (0.0)	0 (0.0)

Note: Data are presented as frequency (percentage). PLA = placebo; CAF3 = 3 mg/kg caffeinated chewing gum; CAF6 = 6 mg/kg caffeinated chewing gum. Blinding guesses were recorded immediately post-visit, whereas adverse effects were assessed 24 h post-visit via a self-report questionnaire.

Blinding responses also differed by condition. Perceived caffeine ingestion was reported in 0/15 participants in PLA (0.0%), 8/15 in CAF3 (53.3%), and 12/15 in CAF6 (80.0%). These responses indicate incomplete participant masking, particularly in CAF6, but do not establish accurate identification of the specific nominal dose.

## 4. Discussion

This study compared 3 and 6 mg/kg caffeinated chewing gum in elite female volleyball players using a repeated blocking fatigue protocol. The corresponding results are presented in Table 2A–2B and Figure 1. The main finding was outcome-specific rather than a uniform dose-related advantage: CAF6 showed a smaller decline in explosive mechanical output and the smallest increase in overall sequence time, whereas CAF3 showed little change in the mean stationary and movement component times. CAF6 showed a smaller descriptive decline in BJ peak power, but the adjusted comparison with CAF3 did not establish superiority. These findings should be interpreted alongside the ISSN position stand and quantitative syntheses [10–12] and the primary volleyball experiments reporting task-specific responses to caffeine [26–30]; caffeine did not prevent the post-fatigue reduction in jump height across conditions. Volleyball studies have also shown that caffeine effects depend on the outcome and the testing context across different test modes and skill constraints [26–30]. The timing-chain results should therefore be interpreted as component-specific rather than as evidence of a general CAF3 timing-stability benefit.

The supplementary models showed condition × time interactions in the primary outcomes, but this did not establish significance for every pairwise dose comparison. The adjusted POST comparison of BJ peak power between CAF6 and CAF3 was not significant (*p* = 0.112; Supplementary Table S3). Because PRE was collected after supplementation, POST differences and differences in PRE-to-POST change provide complementary information. Dose comparisons should therefore be interpreted using the corresponding adjusted contrasts rather than the omnibus interaction alone.

The descriptive mechanical pattern differed between power-related variables and jump height. As shown in Table 3 and Figure 3, jump height declined across conditions, and the reduction in BJ peak power was smaller under CAF6. This pattern may reflect differences in force-time organization under fatigue rather than preservation of jump height itself. Volleyball imposes repeated stretch-shortening cycle loads. Fatigue exposure can disrupt stretch-shortening cycle function and stiffness-related behavior, and athletes often prolong contact or propulsive time to compensate for impaired force generation [47,48]. This pattern was evident in the placebo condition, where DJ contact time increased by 20.0% and BJ take-off time increased markedly. CAF6-related preservation of BJ peak power also aligned with the smaller deterioration observed in DJ vertical stiffness and related SSC measures. This pattern is compatible with altered force transfer under fatigue, although it does not establish superiority of CAF6 over CAF3 for BJ peak power.

However, the CMJ mRSI result should be interpreted separately from the other CMJ/DJ outcomes. Although CMJ mRSI showed a significant Condition × Time interaction, its direction did not support a caffeine-related protective effect. PLA showed an increase in CMJ mRSI despite a reduction in CMJ jump height, whereas CAF3 remained stable and CAF6 decreased slightly. Because CMJ mRSI is derived from both jump height and time to take-off, this pattern may partly reflect the influence of the temporal component rather than superior preservation of reactive strength in PLA. However, because CMJ time to take-off was not reported as a separate outcome, this interpretation remains tentative. Accordingly, CMJ mRSI was interpreted cautiously and was not considered primary evidence for the ergogenic effect.

CAF3 and CAF6 showed smaller fatigue-related prolongation in BJ take-off time based on descriptive changes, although the Condition × Time interaction was not significant. Accordingly, the visual separation seen in BJ take-off time and BJ jump height should be interpreted cautiously and not as confirmatory evidence of statistically supported between-condition differences.

Blocking is a perception–action coupling skill. Players must extract cues quickly and align movement timing precisely. Volleyball perceptual research shows that elite players use more efficient cue utilization and timing strategies under time pressure [49]. CAF3 showed little PRE-to-POST change in the group means for stationary and movement response times. This observation describes preservation of average component durations; it does not establish lower trial-to-trial variability, greater within-athlete consistency, or superior coordination because no such timing-stability metric was prespecified or analyzed. In semi-closed sport skills such as blocking, component response-time patterns may be as practically relevant as gross mechanical output. Accordingly, the CAF3 pattern is interpreted as an outcome-specific observation rather than evidence of a general timing-stability benefit. Evidence also indicates that caffeine can support cognitive and psychomotor components in sport contexts [50,51].

CAF6 showed the largest descriptive reduction in mean stationary BJ response time. The adjusted POST comparisons for stationary response time were not significant (all *p* ≥ 0.150; Supplementary Table S3). Overall sequence time increased by 18.4% in PLA, 6.4% in CAF3, and 3.0% in CAF6; thus CAF6 showed the smallest descriptive deterioration in the integrated timing outcome. These component-specific patterns may reflect arousal-related responses under fatigue. Higher arousal may preferentially improve early response speed, but it may not optimize performance in more complex phases. Other skill studies after caffeine ingestion have reported similar patterns [52]. Fatigue can impair muscular output and central information-processing speed. Fatigue can also impair vigilance-related function [51]. Mental-fatigue evidence also supports impairments in physical performance and cognitive–motor execution under fatigue-like constraints [7,8]. Semi-closed skills operate under strict time constraints. The component outcomes were not identical: CAF3 showed little change in the displayed group means for stationary and movement response times, whereas CAF6 showed the smallest change in overall sequence time. These observations do not support recommending CAF3 as the superior timing condition.

The exploratory neuromuscular results were consistent with the performance pattern but should not be treated as confirmatory evidence. Under CAF6, several extensor-related measures, including VM iEMG and RMS and GM and RF frequency-domain variables, were better preserved after fatigue. CAF3 also showed favorable preservation in selected MF outcomes, particularly in RF, VM, and BF; however, these exploratory EMG patterns do not directly establish timing stability. Fatigue-related neural and metabolic changes can alter the surface EMG spectrum, including frequency-domain indices such as median frequency [53,54]. These patterns are compatible with caffeine-related support of neural drive and neuromuscular function [55–57], but the sEMG analyzes were exploratory and were used only as mechanistic context.

CAF6 also showed shorter muscle activation time in selected muscles. A shorter activation time may reflect greater central drive or altered inhibitory control, and prior evidence suggests that caffeine can influence motor-unit behavior and spinal excitability in humans [58,59]. This mechanism is one possible interpretation of the within-condition reduction in stationary response time under CAF6, but it does not demonstrate superiority over CAF3. However, faster activation does not necessarily translate into better coordination in more complex phases, which may help explain why CAF6 did not consistently outperform CAF3 across the full timing chain.

The blinding and tolerability results warrant cautious interpretation. Perceived caffeine ingestion was reported in 53.3% of CAF3 visits and 80.0% of CAF6 visits, compared with 0.0% of PLA visits. Thus, although randomization and allocation concealment procedures were implemented, participant masking was incomplete, particularly in CAF6. The reported percentages concern perceived caffeine presence and do not establish accurate identification of the specific nominal dose. Sensory differences, such as bitterness, and perceived physiological or stimulatory effects are possible explanations; the present assessment did not separate their contributions. Expectancy effects could have influenced effort, arousal, motivation, and voluntary responses, especially for timing outcomes. Accordingly, the observed condition differences cannot be attributed exclusively to pharmacological dose effects. Adverse effects were also more frequent in CAF6.

Several limitations should be acknowledged. The sample was modest and restricted to elite female volleyball players with low habitual caffeine intake, which limits generalizability to other athletic populations. Menstrual-cycle day was recorded and visits were scheduled within comparable phases when possible, but cycle phase was not formally modeled statistically and may have contributed to residual variability. Because POST tests were completed in a fixed order, recovery intervals differed across outcomes and may have contributed to outcome-specific responses. Supplementary sensitivity analyzes addressed period and treatment sequence for the four primary outcomes, but the small sample limited the precision of sequence-effect estimates. The present study also assessed laboratory proxies for reactive blocking execution rather than match-level blocking success or opponent attack suppression [1,2], and the sEMG findings should therefore be interpreted as exploratory mechanistic support rather than confirmatory evidence. CAF3 and CAF6 are interpreted as nominal label-based target-dose conditions rather than analytically characterized caffeine concentrations because dosing used manufacturer-label-based quarter-piece fractionation. Habitual caffeine intake was classified using the questionnaire-based eligibility threshold rather than a quantitative mg/day or mg/kg/day estimate. Future studies should use analytically characterized whole-gum and quarter-piece dose units, together with source-, portion-, and frequency-specific caffeine records, to quantify administered caffeine and habitual exposure more precisely. Finally, because all participants were low habitual caffeine consumers, the findings should be interpreted within that context and may not generalize directly to athletes with moderate or high habitual caffeine intake, whose acute responsiveness may differ [17,18]. Future studies should test whether the present dose-related pattern is maintained in athletes with moderate or high habitual caffeine intake and should extend the present design to field-based or match-level blocking outcomes, such as block-point success and opponent attack suppression.

Overall, the findings suggest that dose may matter for different components of blocking performance in this sample. CAF6 showed a smaller descriptive decline in BJ peak power and selected stretch-shortening cycle-related characteristics, whereas CAF3 showed little change in selected component response times; the corresponding dose comparisons require outcome-specific interpretation. These observations do not establish a general timing-stability advantage for CAF3. These findings apply to laboratory blocking proxies rather than direct match-level blocking outcomes.

## 5. Conclusion

In elite female volleyball players, caffeinated chewing gum affected blocking-related performance differently across mechanical and timing outcomes after a fatigue-inducing blocking protocol. CAF6 showed a smaller decline in BJ peak power and the smallest increase in overall sequence time, while CAF3 showed little change in mean stationary and movement response times. Direct dose comparisons did not establish a general advantage of CAF6 over CAF3, and the findings do not establish a timing-stability advantage for CAF3 or a universally preferable dose. Interpretation is limited by incomplete participant masking, label-based dose estimation, and questionnaire-based classification of habitual caffeine intake, and the findings apply to the laboratory tasks studied.

## Supplementary Material

Supplementary Material 04Supplementary_Material_clean.docx

## Data Availability

The data that support the findings of this study are available from the corresponding author upon reasonable request. Restrictions apply due to institutional and participant-related confidentiality requirements.

## Trial registration

This study was not prospectively registered.
