# Supplementary material for: Dose-specific effects of caffeinated chewing gum on reactive blocking timing, jump mechanics, and neuromuscular responses after volleyball-specific fatigue in elite female volleyball players
Source: J Int Soc Sports Nutr. 2026 Nov 9;23(1):2749890. doi: 10.1080/15502783.2026.2749890 (PMC13757810; doi:10.1080/15502783.2026.2749890)
Supplement: Supplementary file 1 [file RSSN_A_2749890_SM3295.docx]

**Supplementary Material**

**Table S1. Treatment Sequence Distribution**

| **Treatment sequence** | **Period 1** | **Period 2** | **Period 3** | **n** |
| --- | --- | --- | --- | --- |
| PLA → CAF3 → CAF6 | PLA | CAF3 | CAF6 | 3 |
| PLA → CAF6 → CAF3 | PLA | CAF6 | CAF3 | 2 |
| CAF3 → PLA → CAF6 | CAF3 | PLA | CAF6 | 3 |
| CAF3 → CAF6 → PLA | CAF3 | CAF6 | PLA | 2 |
| CAF6 → PLA → CAF3 | CAF6 | PLA | CAF3 | 3 |
| CAF6 → CAF3 → PLA | CAF6 | CAF3 | PLA | 2 |
| Total |  |  |  | 15 |

Note. Periods 1–3 denote the chronological order of the formal experimental visits. Each participant completed PLA, CAF3, and CAF6 once. PLA = placebo; CAF3 and CAF6 = nominal caffeine doses of 3 and 6 mg/kg, respectively.

**Table S2. Period and Treatment Sequence Sensitivity Analysis and PRE Condition Comparisons**

| **Outcome** | **Model term** | **F** | **Numerator df** | **Denominator df** | **p** |
| --- | --- | --- | --- | --- | --- |
| BJ peak power | Condition × Time | 4.42 | 2 | 26.8 | 0.022 |
|  | Period | 0.41 | 2 | 27.6 | 0.668 |
|  | Period × Time | 0.36 | 2 | 25.9 | 0.702 |
|  | Treatment sequence | 0.58 | 5 | 8.7 | 0.715 |
| Stationary BJ response time | Condition × Time | 24.80 | 2 | 27.2 | <0.001 |
|  | Period | 0.52 | 2 | 26.5 | 0.600 |
|  | Period × Time | 0.44 | 2 | 27.9 | 0.648 |
|  | Treatment sequence | 0.62 | 5 | 8.9 | 0.689 |
| Movement BJ response time | Condition × Time | 32.60 | 2 | 26.4 | <0.001 |
|  | Period | 0.63 | 2 | 28.1 | 0.540 |
|  | Period × Time | 0.48 | 2 | 27.0 | 0.624 |
|  | Treatment sequence | 0.71 | 5 | 9.2 | 0.631 |
| Overall sequence time | Condition × Time | 26.70 | 2 | 27.1 | <0.001 |
|  | Period | 0.52 | 2 | 26.7 | 0.600 |
|  | Period × Time | 0.44 | 2 | 28.4 | 0.648 |
|  | Treatment sequence | 0.62 | 5 | 8.8 | 0.689 |
| Panel B. Condition comparisons at PRE | | | | | |
| BJ peak power | Condition at PRE | 0.08 | 1.331 | 18.633 | 0.853 |
| Stationary BJ response time | Condition at PRE | 11.71 | 1.591 | 22.268 | <0.001 |
| Movement BJ response time | Condition at PRE | 27.29 | 1.972 | 27.602 | <0.001 |
| Overall sequence time | Condition at PRE | 36.48 | 1.655 | 23.166 | <0.001 |

Note. Panel A presents F tests from supplementary mixed-effects analyses accounting for repeated observations within participants. Period denotes chronological experimental visit, and treatment sequence denotes the assigned order of PLA, CAF3, and CAF6. Panel B presents separate PRE condition comparisons using the three-trial means of 15 participants. Panel B uses repeated-measures ANOVA with Greenhouse–Geisser-corrected degrees of freedom and p-values and is not adjusted for period or sequence. df = degrees of freedom.

**Table S3. Period- and Sequence-Adjusted Treatment Comparisons for the Four Primary Outcomes**

| **Outcome** | **Estimand** | **Comparison** | **Adjusted estimate** | **95% CI** | **p** |
| --- | --- | --- | --- | --- | --- |
| BJ peak power (W) | POST difference | CAF3 − PLA | 84.5 | −73.2 to 242.1 | 0.270 |
|  | POST difference | CAF6 − PLA | 209.3 | 51.6 to 366.9 | 0.013 |
|  | POST difference | CAF6 − CAF3 | 124.8 | −32.8 to 282.4 | 0.112 |
|  | Change difference | CAF3 − PLA | 68.4 | −117.6 to 254.4 | 0.443 |
|  | Change difference | CAF6 − PLA | 229.9 | 43.9 to 415.9 | 0.019 |
|  | Change difference | CAF6 − CAF3 | 161.5 | −24.5 to 347.5 | 0.084 |
| Stationary BJ response time (s) | POST difference | CAF3 − PLA | −0.048 | −0.183 to 0.087 | 0.459 |
|  | POST difference | CAF6 − PLA | −0.096 | −0.231 to 0.039 | 0.150 |
|  | POST difference | CAF6 − CAF3 | −0.048 | −0.183 to 0.087 | 0.459 |
|  | Change difference | CAF3 − PLA | −0.162 | −0.321 to −0.002 | 0.047 |
|  | Change difference | CAF6 − PLA | −0.257 | −0.416 to −0.097 | 0.004 |
|  | Change difference | CAF6 − CAF3 | −0.095 | −0.254 to 0.064 | 0.222 |
| Movement BJ response time (s) | POST difference | CAF3 − PLA | −0.298 | −0.433 to −0.162 | <0.001 |
|  | POST difference | CAF6 − PLA | −0.192 | −0.327 to −0.057 | 0.009 |
|  | POST difference | CAF6 − CAF3 | 0.106 | −0.030 to 0.241 | 0.116 |
|  | Change difference | CAF3 − PLA | −0.541 | −0.701 to −0.382 | <0.001 |
|  | Change difference | CAF6 − PLA | −0.418 | −0.577 to −0.259 | <0.001 |
|  | Change difference | CAF6 − CAF3 | 0.123 | −0.036 to 0.283 | 0.119 |
| Overall sequence time (s) | POST difference | CAF3 − PLA | −0.422 | −0.558 to −0.287 | <0.001 |
|  | POST difference | CAF6 − PLA | −0.605 | −0.740 to −0.470 | <0.001 |
|  | POST difference | CAF6 − CAF3 | −0.182 | −0.318 to −0.047 | 0.012 |
|  | Change difference | CAF3 − PLA | −0.712 | −0.872 to −0.553 | <0.001 |
|  | Change difference | CAF6 − PLA | −0.931 | −1.090 to −0.772 | <0.001 |
|  | Change difference | CAF6 − CAF3 | −0.218 | −0.378 to −0.059 | 0.011 |

Note. Estimates are adjusted between-condition differences from the sensitivity models. PRE denotes post-supplementation, pre-fatigue testing, and POST denotes post-fatigue testing. For a comparison A − B, the change difference is calculated as (POST − PRE)A − (POST − PRE)B. BJ peak power is expressed in W, and timing outcomes are expressed in s. CI = confidence interval.

| **Table S4. Complete integrated EMG (iEMG) results for all eight recorded muscles during the block jump task** | | | | | | |
| --- | --- | --- | --- | --- | --- | --- |
| **Muscle** | **Condition** | **PRE** | **POST** | **Time × Condition F** | ***p*** | **ηp²** |
| GM | PLA | 1.43 ± 1.65 | 1.52 ± 1.77^***^ | 26.10 | <0.001 | 0.667 |
|  | CAF3 | 1.81 ± 2.09 | 1.56 ± 1.89 |  |  |  |
|  | CAF6 | 1.17 ± 1.21 | 1.24 ± 1.39^***^ |  |  |  |
| RF | PLA | 0.76 ± 0.25 | 0.72 ± 0.23^***^ | 19.77 | <0.001 | 0.603 |
|  | CAF3 | 0.82 ± 0.37 | 0.77 ± 0.30 |  |  |  |
|  | CAF6 | 0.82 ± 0.33 | 0.80 ± 0.26 |  |  |  |
| VM | PLA | 0.85 ± 0.24 | 0.72 ± 0.19^***^ | 318.05 | <0.001 | 0.961 |
|  | CAF3 | 0.84 ± 0.23 | 0.75 ± 0.17^***^ |  |  |  |
|  | CAF6 | 0.85 ± 0.36 | 0.85 ± 0.31 |  |  |  |
| VL | PLA | 1.35 ± 0.34 | 1.15 ± 0.27^***^ | 210.10 | <0.001 | 0.942 |
|  | CAF3 | 1.26 ± 0.37 | 1.19 ± 0.30^***^ |  |  |  |
|  | CAF6 | 1.48 ± 0.42 | 1.50 ± 0.35^***^ |  |  |  |
| BF | PLA | 0.36 ± 0.11 | 0.39 ± 0.11^***^ | 42.93 | <0.001 | 0.768 |
|  | CAF3 | 0.35 ± 0.13 | 0.36 ± 0.11 |  |  |  |
|  | CAF6 | 0.35 ± 0.14 | 0.31 ± 0.11^***^ |  |  |  |
| ST | PLA | 0.27 ± 0.10 | 0.28 ± 0.11^***^ | 17.24 | <0.001 | 0.570 |
|  | CAF3 | 0.27 ± 0.14 | 0.26 ± 0.11 |  |  |  |
|  | CAF6 | 0.28 ± 0.12 | 0.26 ± 0.10^***^ |  |  |  |
| MG | PLA | 0.64 ± 0.20 | 0.56 ± 0.17^***^ | 50.49 | <0.001 | 0.795 |
|  | CAF3 | 0.63 ± 0.27 | 0.61 ± 0.20^***^ |  |  |  |
|  | CAF6 | 0.69 ± 0.32 | 0.64 ± 0.30 |  |  |  |
| TA | PLA | 2.14 ± 2.50 | 2.15 ± 2.51 | 1.54 | 0.234 | 0.106 |
|  | CAF3 | 2.34 ± 3.05 | 2.06 ± 2.61 |  |  |  |
|  | CAF6 | 2.12 ± 2.31 | 2.30 ± 2.82^*^ |  |  |  |
|  |  |  |  |  |  |  |
| Note. Data are presented as mean ± SD in normalized arbitrary units (a.u.). For each muscle, normalization used the maximum valid MVC reference obtained at the same experimental visit before performance testing; the same reference was applied to PRE and POST. PRE = post-supplementation, pre-fatigue; POST = post-fatigue; PLA = placebo; CAF3 = 3 mg/kg caffeinated chewing gum; CAF6 = 6 mg/kg caffeinated chewing gum.  * p < 0.05 and *** p < 0.001 indicate significant within-condition differences between PRE and POST.  GM = gluteus maximus; RF = rectus femoris; VM = vastus medialis; VL = vastus lateralis; BF = biceps femoris; ST = semitendinosus; MG = medial gastrocnemius; TA = tibialis anterior. | | | | | | |

| **Table S5. Complete root mean square (RMS) results for all eight recorded muscles during the block jump task** | | | | | | |
| --- | --- | --- | --- | --- | --- | --- |
| **Muscle** | **Condition** | **PRE** | **POST** | **Time × Condition F** | ***p*** | **ηp²** |
| GM | PLA | 0.78 ± 0.38 | 0.83 ± 0.42^***^ | 0.26 | 0.776 | 0.019 |
|  | CAF3 | 0.92 ± 1.00 | 0.91 ± 0.39 |  |  |  |
|  | CAF6 | 0.87 ± 1.11 | 0.82 ± 0.46^*^ |  |  |  |
| RF | PLA | 1.04 ± 0.27 | 0.97 ± 0.23^***^ | 25.72 | <0.001 | 0.664 |
|  | CAF3 | 1.12 ± 0.60 | 1.08 ± 0.41^*^ |  |  |  |
|  | CAF6 | 0.99 ± 0.47 | 0.94 ± 0.41^*^ |  |  |  |
| VM | PLA | 1.26 ± 0.35 | 1.07 ± 0.30^***^ | 67.32 | <0.001 | 0.838 |
|  | CAF3 | 1.32 ± 0.65 | 1.21 ± 0.46^*^ |  |  |  |
|  | CAF6 | 1.39 ± 0.65 | 1.45 ± 0.50^***^ |  |  |  |
| VL | PLA | 2.05 ± 0.58 | 1.75 ± 0.49^***^ | 27.90 | <0.001 | 0.682 |
|  | CAF3 | 1.74 ± 0.96 | 1.65 ± 0.69^***^ |  |  |  |
|  | CAF6 | 1.81 ± 0.91 | 1.92 ± 0.90^***^ |  |  |  |
| BF | PLA | 0.58 ± 0.22 | 0.62 ± 0.24^***^ | 0.00 | 0.996 | 0.000 |
|  | CAF3 | 0.62 ± 0.62 | 0.64 ± 0.30^*^ |  |  |  |
|  | CAF6 | 0.68 ± 1.00 | 0.59 ± 0.31 |  |  |  |
| ST | PLA | 0.41 ± 0.16 | 0.43 ± 0.18^***^ | 0.40 | 0.677 | 0.030 |
|  | CAF3 | 0.41 ± 0.65 | 0.45 ± 0.52 |  |  |  |
|  | CAF6 | 0.32 ± 0.93 | 0.31 ± 0.23 |  |  |  |
| MG | PLA | 0.95 ± 0.28 | 0.83 ± 0.25^***^ | 4.04 | 0.030 | 0.237 |
|  | CAF3 | 1.03 ± 0.84 | 1.00 ± 0.32 |  |  |  |
|  | CAF6 | 1.28 ± 0.89 | 1.21 ± 0.59 |  |  |  |
| TA | PLA | 1.00 ± 0.42 | 1.00 ± 0.42 | 2.30 | 0.121 | 0.150 |
|  | CAF3 | 1.11 ± 0.77 | 1.04 ± 0.55 |  |  |  |
|  | CAF6 | 1.18 ± 0.88 | 1.13 ± 0.73 |  |  |  |
| Note. Data are presented as mean ± SD in normalized arbitrary units (a.u.). For each muscle, normalization used the maximum valid MVC reference obtained at the same experimental visit before performance testing; the same reference was applied to PRE and POST. PRE = post-supplementation, pre-fatigue; POST = post-fatigue; PLA = placebo; CAF3 = 3 mg/kg caffeinated chewing gum; CAF6 = 6 mg/kg caffeinated chewing gum.  * p < 0.05 and *** p < 0.001 indicate significant within-condition differences between PRE and POST.  GM = gluteus maximus; RF = rectus femoris; VM = vastus medialis; VL = vastus lateralis; BF = biceps femoris; ST = semitendinosus; MG = medial gastrocnemius; TA = tibialis anterior. | | | | | | |

| **Table S6. Complete median frequency (MF) results for all eight recorded muscles during the block jump task** | | | | | | |
| --- | --- | --- | --- | --- | --- | --- |
| **Muscle** | **Condition** | **PRE** | **POST** | **Time × Condition F** | ***p*** | **ηp²** |
| GM | PLA | 85.8 ± 35.2 | 77.3 ± 31.3^***^ | 214.20 | <0.001 | 0.943 |
|  | CAF3 | 83.9 ± 34.7 | 78.7 ± 27.9^***^ |  |  |  |
|  | CAF6 | 85.3 ± 39.6 | 89.4 ± 40.4 |  |  |  |
| RF | PLA | 88.6 ± 12.9 | 79.8 ± 11.1^***^ | 196.08 | <0.001 | 0.938 |
|  | CAF3 | 91.4 ± 20.1 | 86.4 ± 12.6^***^ |  |  |  |
|  | CAF6 | 86.6 ± 16.6 | 86.4 ± 10.4 |  |  |  |
| VM | PLA | 82.0 ± 10.4 | 73.8 ± 7.8^**^ | 90.37 | <0.001 | 0.874 |
|  | CAF3 | 82.8 ± 17.6 | 79.1 ± 11.5^**^ |  |  |  |
|  | CAF6 | 83.3 ± 19.2 | 81.7 ± 14.4 |  |  |  |
| VL | PLA | 81.9 ± 12.0 | 73.8 ± 10.3^***^ | 333.09 | <0.001 | 0.962 |
|  | CAF3 | 76.1 ± 16.4 | 73.6 ± 11.2^***^ |  |  |  |
|  | CAF6 | 77.4 ± 15.6 | 76.8 ± 15.3^**^ |  |  |  |
| BF | PLA | 85.2 ± 15.1 | 76.9 ± 13.1^***^ | 183.11 | <0.001 | 0.934 |
|  | CAF3 | 85.6 ± 21.1 | 81.7 ± 11.9^***^ |  |  |  |
|  | CAF6 | 80.8 ± 18.8 | 82.0 ± 17.2 |  |  |  |
| ST | PLA | 79.2 ± 15.2 | 71.3 ± 13.3^***^ | 188.55 | <0.001 | 0.936 |
|  | CAF3 | 79.0 ± 21.2 | 74.8 ± 15.3^***^ |  |  |  |
|  | CAF6 | 82.9 ± 20.1 | 83.7 ± 17.9 |  |  |  |
| MG | PLA | 129.4 ± 11.9 | 116.1 ± 10.7^***^ | 235.62 | <0.001 | 0.948 |
|  | CAF3 | 133.6 ± 18.5 | 126.1 ± 14.0^***^ |  |  |  |
|  | CAF6 | 130.0 ± 24.3 | 128.4 ± 20.8 |  |  |  |
| TA | PLA | 107.8 ± 32.0 | 97.2 ± 28.4^***^ | 155.59 | <0.001 | 0.923 |
|  | CAF3 | 104.2 ± 38.3 | 101.7 ± 31.8^***^ |  |  |  |
|  | CAF6 | 108.7 ± 36.6 | 106.9 ± 31.1^**^ |  |  |  |
| Note. Data are presented as mean ± SD. PRE = post-supplementation, pre-fatigue; POST = post-fatigue; PLA = placebo; CAF3 = 3 mg/kg caffeinated chewing gum; CAF6 = 6 mg/kg caffeinated chewing gum.  * p < 0.05, ** p < 0.01, and *** p < 0.001 indicate significant within-condition differences between PRE and POST.  GM = gluteus maximus; RF = rectus femoris; VM = vastus medialis; VL = vastus lateralis; BF = biceps femoris; ST = semitendinosus; MG = medial gastrocnemius; TA = tibialis anterior. | | | | | | |

| **Table S7. Complete muscle activation time (MAT) results for all eight recorded muscles during the block jump task** | | | | | | |
| --- | --- | --- | --- | --- | --- | --- |
| **Muscle** | **Condition** | **PRE** | **POST** | **Time × Condition F** | ***p*** | **ηp²** |
| GM | PLA | 0.19 ± 0.11 | 0.21 ± 0.10^***^ | 18.63 | <0.001 | 0.589 |
|  | CAF3 | 0.17 ± 0.20 | 0.18 ± 0.15 |  |  |  |
|  | CAF6 | 0.16 ± 0.19 | 0.14 ± 0.15^*^ |  |  |  |
| RF | PLA | 0.15 ± 0.10 | 0.17 ± 0.10^***^ | 4.10 | 0.028 | 0.240 |
|  | CAF3 | 0.18 ± 0.19 | 0.18 ± 0.16 |  |  |  |
|  | CAF6 | 0.14 ± 0.17 | 0.12 ± 0.12^**^ |  |  |  |
| VM | PLA | 0.12 ± 0.07 | 0.13 ± 0.07^***^ | 4.42 | 0.022 | 0.254 |
|  | CAF3 | 0.12 ± 0.18 | 0.13 ± 0.13 |  |  |  |
|  | CAF6 | 0.16 ± 0.19 | 0.15 ± 0.12^**^ |  |  |  |
| VL | PLA | 0.13 ± 0.09 | 0.14 ± 0.09^***^ | 1.43 | 0.259 | 0.099 |
|  | CAF3 | 0.11 ± 0.18 | 0.13 ± 0.15 |  |  |  |
|  | CAF6 | 0.10 ± 0.20 | 0.11 ± 0.15^*^ |  |  |  |
| BF | PLA | 0.22 ± 0.13 | 0.24 ± 0.16^***^ | 4.36 | 0.023 | 0.251 |
|  | CAF3 | 0.23 ± 0.24 | 0.24 ± 0.17 |  |  |  |
|  | CAF6 | 0.17 ± 0.23 | 0.13 ± 0.14 |  |  |  |
| ST | PLA | 0.20 ± 0.12 | 0.22 ± 0.15^***^ | 27.66 | <0.001 | 0.680 |
|  | CAF3 | 0.15 ± 0.21 | 0.15 ± 0.12^*^ |  |  |  |
|  | CAF6 | 0.16 ± 0.24 | 0.14 ± 0.23 |  |  |  |
| MG | PLA | 0.21 ± 0.15 | 0.23 ± 0.18^***^ | 2.67 | 0.088 | 0.171 |
|  | CAF3 | 0.21 ± 0.32 | 0.22 ± 0.28 |  |  |  |
|  | CAF6 | 0.17 ± 0.26 | 0.14 ± 0.14^*^ |  |  |  |
| TA | PLA | 0.04 ± 0.06 | 0.05 ± 0.07^**^ | 1.64 | 0.213 | 0.112 |
|  | CAF3 | 0.05 ± 0.10 | 0.05 ± 0.13 |  |  |  |
|  | CAF6 | 0.03 ± 0.10 | 0.02 ± 0.07* |  |  |  |
| Note. Data are presented as mean ± SD. PRE = post-supplementation, pre-fatigue; POST = post-fatigue; PLA = placebo; CAF3 = 3 mg/kg caffeinated chewing gum; CAF6 = 6 mg/kg caffeinated chewing gum.  * p < 0.05, ** p < 0.01, and *** p < 0.001 indicate significant within-condition differences between PRE and POST.  GM = gluteus maximus; RF = rectus femoris; VM = vastus medialis; VL = vastus lateralis; BF = biceps femoris; ST = semitendinosus; MG = medial gastrocnemius; TA = tibialis anterior. | | | | | | |
